# Tau as a mediator of neurotoxicity associated to cerebral amyloid angiopathy

**DOI:** 10.1186/s40478-019-0680-z

**Published:** 2019-02-26

**Authors:** Yingjian You, Abigail Perkins, Pablo Cisternas, Braulio Muñoz, Xavier Taylor, Yanwen You, Holly J. Garringer, Adrian L. Oblak, Brady K. Atwood, Ruben Vidal, Cristian A. Lasagna-Reeves

**Affiliations:** 1 Stark Neurosciences Research Institute, Indiana University School of Medicine, Indianapolis, IN 46202 USA; 2 Department of Anatomy & Cell Biology, Indiana University School of Medicine, Indianapolis, IN 46202 USA; 3 Department of Psychiatry, Indiana University School of Medicine, Indianapolis, IN 46202 USA; 4 Department of Pathology and Laboratory Medicine, Indiana University School of Medicine, Indianapolis, IN 46202 USA; 5 Indiana Alzheimer Disease Center, Indiana University School of Medicine, Indianapolis, IN 46202 USA; 6 Department of Radiology & Imaging Sciences, Indiana University School of Medicine, Indianapolis, IN 46202 USA; 7 Department of Pharmacology & Toxicology, Indiana University School of Medicine, Indianapolis, IN 46202 USA; 8 Indiana University School of Medicine, The Stark Neurosciences Research Institute, Neurosciences Research Building 214G, 320 West 15th Street, Indianapolis, IN 46202 USA

**Keywords:** Cerebral amyloid angiopathy, Tau oligomers, ADan oligomers, Vascular amyloid, Tau downregulation, Neurodegeneration

## Abstract

Cerebral amyloid angiopathy (CAA) is typified by the cerebrovascular deposition of amyloid. Currently, there is no clear understanding of the mechanisms underlying the contribution of CAA to neurodegeneration. Despite the fact that CAA is highly associated with accumulation of Aβ, other types of amyloids have been shown to associate with the vasculature. Interestingly, in many cases, vascular amyloidosis is accompanied by significant tau pathology. However, the contribution of tau to neurodegeneration associated to CAA remains to be determined. We used a mouse model of Familial Danish Dementia (FDD), a neurodegenerative disease characterized by the accumulation of Danish amyloid (ADan) in the vasculature, to characterize the contribution of tau to neurodegeneration associated to CAA. We performed histological and biochemical assays to establish tau modifications associated with CAA in conjunction with cell-based and electrophysiological assays to determine the role of tau in the synaptic dysfunction associated with ADan. We demonstrated that ADan aggregates induced hyperphosphorylation and misfolding of tau. Moreover, in a mouse model for CAA, we observed tau oligomers closely associated to astrocytes in the vicinity of vascular amyloid deposits. We finally determined that the absence of tau prevents synaptic dysfunction induced by ADan oligomers. In addition to demonstrating the effect of ADan amyloid on tau misfolding, our results provide compelling evidence of the role of tau in neurodegeneration associated with ADan-CAA and suggest that decreasing tau levels could be a feasible approach for the treatment of CAA.

**Electronic supplementary material:**

The online version of this article (10.1186/s40478-019-0680-z) contains supplementary material, which is available to authorized users.

## Introduction

Alzheimer disease (AD), the most common form of dementia, is characterized by the extracellular deposition of parenchymal and vascular β-amyloid (Aβ), intracellular accumulation of tau as neurofibrillary tangles (NFTs), neuronal cell loss, and significant inflammation [15, 39]. Over the past decades, a major focus of research has been the understanding of the connection between parenchymal Aβ, NFT, and neurodegeneration, while the contribution of vascular pathology to NFT and neurodegeneration remains understudied.

Cerebral amyloid angiopathy (CAA) is typified by the cerebrovascular deposition of amyloid and has a close molecular relationship with AD, but remains clinically distinct. Vascular amyloid accumulation is identified in an estimated 85–95% of individuals with AD [3, 6], positioning CAA as one of the strongest vascular contributors to age-related cognitive decline [9, 64]. The mechanisms responsible for CAA pathogenesis and its downstream effects on the brain are complex and not completely understood [12, 66]. Despite the fact that CAA is highly associated with the accumulation of Aβ [6], other types of amyloids have been shown to associate with the vasculature. It has also been reported that some mutations in the PrP gene may result in the deposition of prion amyloid (APrP) in cerebral vessels (PrP-CAA) [24]. Furthermore, one of the main neuropathological hallmarks of Familial British Dementia (FBD) and Familial Danish Dementia (FDD) is the presence of CAA composed of British-amyloid (ABri) and Danish amyloid (ADan) respectively [22, 61, 63]. These observations suggest that CAA is a general term that describes a heterogeneous group of biochemically and genetically diverse central nervous system disorders, characterized by the dynamic accumulation of different amyloid species in the vasculature.

In many cases, vascular amyloidosis is accompanied by significant tau pathology [24, 49, 60]. Although different amyloid peptides are deposited in these conditions, the tau deposits are antigenically and biochemically indistinguishable [25, 27, 28, 34, 35]. These findings support a unifying pathological mechanism in which vascular accumulation of amyloidogenic peptides triggers a complex pathological cascade leading to tau accumulation and neurodegeneration.

FDD patients are characterized by the presence of CAA composed of the 4 kDa ADan in leptomeninges and vessels of the gray and white matter. Genetic analysis in patients with FDD revealed the presence of a 10-nucleotide duplication insertion in the 3′-end of the coding region of the *BRI2* gene. The mutation in *BRI2* causes a frame-shift in the BRI2 sequence, generating a ADan precursor protein of 277 amino acids, of which the ~ 4 kDa Danish amyloid subunit comprises the last 34 amino acids [22]. Cotton wool-like plaques in the vicinity of blood vessels with amyloid and tau NFTs are also observed in FDD patients [34]. A mouse model for Familial Danish Dementia (Tg-FDD) [59] consistently exhibits CAA primarily in leptomeningeal cerebellar vessels [59] and in large and medium-sized parenchymal and penetrating vessels of the brain. Neuropathologically, a robust glial activation is observed in close vicinity of vascular deposits without the presence of cerebral hemorrhage [59]. Tau immunoreactive deposits in neuropil have also been observed in this model [59], yet the spatial relationship between vascular amyloid deposits and tau in Tg-FDD mice has not been established. Overall these observations make FDD and the Tg-FDD mice a valuable model to study the molecular and cellular mechanisms underlying the role of tau in the neurodegenerative process associated with CAA.

In the present study, we show that ADan induced the phosphorylation and misfolding of tau and subsequent tau-dependent neurotoxicity. Our results suggest that ADan aggregates could have an effect over tau via two different and non-excluding pathways, and how the absence of tau could prevent the synaptic dysfunction induced by CAA-associated amyloid.

## Materials and methods

### ADan oligomers preparation

ADan peptide (EASNCFAIRHFENKFAVETLICFNLFLNSQEKHY) [63] was synthesized by ThermoFisher Scientific using Fmoc-based Solid Phase Peptide Synthesis and purified by HPLC. To prepare ADan oligomers, ADan peptide was resuspended in PBS without calcium and magnesium to a final concentration of 0.5 mg/mL. The ADan solution was then stirred at room temperature (RT) for 48 h. Aliquots were collected at different time points and stored at − 80 °C. To confirm the formation of ADan oligomers, Western Blot (WB) analysis was performed using anti-ADan 1699 antibody (1:1000, developed by R. Vidal) that was specific for residues 23–34 (FNLFLNSQEKHY) of the ADan amyloid peptide [59] and the conformational antibody anti-oligomers F11G3 (1:1000, provided by R. Kayed), as previously described [44].

### Cell culture, transfection, and oligomers treatment

Human embryonic kidney cells expressing doxycycline (Dox) inducible full length human tau (2N4R) with the P301L mutation (HEK P301L) ([16] and Additional file 1: Figure S1) were cultured in DMEM (Invitrogen) with 10% fetal bovine serum (Invitrogen), 2 mM glutamine, 100 U/mL penicillin, and 100 μg/mL streptomycin. 1 μg/mL Dox was added into the culture media to induce human tau expression 24 h prior to transfection and ADan oligomer treatment. Human wild-type (WT) BRI_2_ and BRI_2_ bearing the Danish mutation were cloned into a pcDNA3.1 vector (Invitrogen). The sequences were confirmed by Sanger sequencing. All plasmids were transfected with Lipofectamine 2000 (Invitrogen) and incubated for 48 h. For ADan oligomer treatment, human Tau expression was induced by 1 μg/mL Dox in HEK P301L cells for 24 h, the cells were treated with 200 nM ADan oligomers, monomers, or PBS for 8 h. All measurements were made in triplicates.

### Cell toxicity assay

HEK P301L cells were plated at 20,000 cells per well in 96-well plates with 1 μg/mL Dox for 24 h. Then the cells were treated with 2.5 μM ADan oligomers, monomers, or PBS. After incubation for 4 h at 37 °C, cell viability was assayed using a 3-(4,5-dimethylthiazol-2-yl)-2,5-diphenyltetrazolium bromide (MTT) cell proliferation assay kit (Promega) according to the manufacturer’s specifications. The MTT assay is a colorimetric assay for assessing cell metabolic activity, which reflects the number of viable cells. All measurements were made in six replicates.

### Cell lysate preparation and immunoblot analysis

Cells were washed twice with 0.5 mL PBS and lysed on ice for 60 min in 80 μL T-PER tissue protein extraction reagent (ThermoFisher Scientific) supplemented with Complete protease inhibitors cocktail (Roche). The cell lysates were then centrifuged at 14,800 rpm for 15 min at 4 °C, the supernatants were analyzed by WB as previously described [44]. Primary antibodies used were anti-tau (tau-5, 1:1500, Abcam), anti-ptau Thr231 (1:1500, Millipore), PHF1 anti-ptau Ser396/Ser404 (1:1000, provided by Dr. Peter Davies), Anti-BRI_2_ (1:100, Santa Cruz Biotech) and Anti-GAPDH (1:10000, Sigma-Aldrich). Tau oligomers levels were measured using the anti-tau oligomer antibody T22 (1:1000, provided by Dr. Rakez Kayed) as previously described [43].

### Immunocytochemistry

HEK P301L cells were plated on the 12 mm Poly-L-Lysine coated coverslips (Corning BioCoat) in 24-well plates at a density of 62,500 cells per well with 1 μg/mL Dox for 24 h, then cells were transfected with BRI_2_ bearing the Danish mutation for 48 h. Then, the coverslips were briefly rinsed with warm TBS and fixed with 2% paraformaldehyde for 10 min at RT, then permeabilized for 5 min with 0.1% Triton X-100. The samples were blocked for 1 h in 5% goat serum and incubated overnight (O.N.) at 4 °C with mouse anti-ptau Thr231 (1:1000, Millipore) and rabbit anti-ADan antibody 1699 (1:500). Cells were then washed in TBS, then incubated with Alexa 488-conjugated goat anti-mouse antibody (1:700, Invitrogen) and Alexa 568-conjugated goat anti-rabbit antibody (1:700, Invitrogen) for 1 h at RT, then washed with TBS and mounted with Vectashield mounting medium with DAPI (Vector Laboratories). Samples were examined using a Leica DMi 8 epifluorescence microscope coupled with the LAS X program (Leica). For orthogonal images of reconstructed three-dimensional views a Nikon A1-R Laser Scanning Confocal Microscope coupled with the NIS Element Advanced Research software was utilized.

### Transgenic mouse model

Tg-FDD, wild type C57/BL6J (WT) (JAX stock #000664) and Tau knock out (Tau^−/−^) (JAX stock #007251) male and female mice were used for our experiments, including cellular, biochemical, and immunohistochemistry (IHC) analyses. The Tg-FDD mouse model [59] expresses a FDD-associated human mutant BRI_2_ transgene. Mice were housed at the Indiana University School of Medicine (IUSM) animal care facility and were maintained according to USDA standards (12-h light/dark cycle, food and water ad libitum), in accordance with the Guide for the Care and Use of Laboratory Animals (National Institutes of Health, Bethesda, MD). Animals were anesthetized and euthanized according to IUSM Institutional Animal Care and Use Committee-approved procedures. Tissue was collected from 3 and 18 months old animals (5 mice per genotype). Mice were deeply anesthetized and perfused transcardially with PBS prior to decapitation. After sacrifice, brains were removed and stored at − 80 °C or formalin fixed as previously described [59].

### Mouse brain samples preparation and immunoblot analysis

Tg-FDD and WT (5 mice per genotype) brains were homogenized at a 1:10 (w/vol) ratio of brain and T-PER tissue protein extraction reagent with complete protease inhibitor cocktail (Roche). Samples were then centrifuged at 13,200 rpm for 15 min at 4 °C. The supernatants were aliquoted, snap-frozen, and stored at − 80 °C until analyzed. The T-PER insoluble pellets were resuspended in 88% formic acid (FA) at one fourth volume of their brain homogenates, then incubated for 1 h at RT. Samples were then diluted with distilled water to obtain the same volume used for brain homogenates. Samples were then lyophilized for 24 h. Freeze-dried samples were reconstituted in PBS using the same volume as brain homogenates, then sonicated for 30 s. Finally, samples were mixed with sample loading buffer, run on a NuPAGE 4–12% Bis-Tris protein gel (Invitrogen), and analyzed by WB. Primary antibodies used were anti-BRI_2_ (1:100, Santa Cruz Biotech), Tau-5 (1:1500, Abcam), PHF1 (anti-ptau Ser396/Ser404) (1:1000, gift from Peter Davies), anti-ptau Thr231 (1:1500, Abcam), MC1 (1:100, gift from Peter Davies), and anti-Vinculin (1:10000, Invitrogen). Secondary antibodies used: goat anti-mouse HRP IgG (1:1500, Invitrogen) and goat anti-rabbit HRP IgG (1:1500, Invitrogen). WB quantification results are expressed as the ratio of phospho-tau to total tau levels. Then this value was normalized by the loading control. In all cases, we considered the control group as the 100%. For the FA treated samples, equal amount of proteins were loaded, previously determined by Bradford assay (ThermoFisher Scientific) according to manufacturer’s specification.

### Brain sections immunofluorescence (IF)

Paraffin sections were deparaffinized in xylene and rehydrated in ethanol (EtOH) and washed with deionized water. Then the sections were heated with a microwave oven in low pH antigen retrieval solution (eBioscience) twice for 4 min each. After washing in TBS twice for 5 min each, the sections were blocked in 5% normal goat serum for 1 h at RT, sections were incubated O.N. at 4 °C with the following antibodies: anti-ADan 1699 (1:500), T22 (1:100, gift from Dr. Rakez Kayed), TOMA (1:750, gift from Dr. Rakez Kayed), MC1 (1:750, gift from Dr. Peter Davies), anti-GFAP antibody (1:200, Sigma-Aldrich), anti-Iba1 (1:250, Millipore), and NeuN (1:100, Abcam). For amyloid detection, sections were first stained with 1% Thioflavin S (Thio-S) in TBS for 8 min before the primary antibody incubation, then washed in EtOH and deionized water. The next day, the sections were washed in TBS and incubated with secondary antibodies Alexa 488-conjugated goat anti-mouse antibody (1:500, Invitrogen) and Alexa 568-conjugated goat anti-rabbit antibody (1:500, Invitrogen) for 1 h at RT. Finally, sections were washed in TBS and mounted with Vectashield mounting medium with DAPI (Vector Laboratories). For colocalization analysis we utilized the Coloc 2 plugin from Fiji that implements and performs the pixel intensity correlation over space methods of Pearson [55]. For every single staining, as a negative control, primary antibodies were omitted to determine background and autofluorescence (not shown). WT and Tg-FDD cerebral cortex was examined using a Leica DMi 8 epifluorescence microscope coupled with the LAS X program (Leica).

### Quantitative PCR

Mouse brains total RNA were isolated with RNeasy Plus Universal Mini kit (Qiagen). cDNA was prepared from 1 μg total RNA with High-Capacity cDNA reverse transcription kit (Life Technologies). All qPCRs were performed on QuantStudio 6 Flex Real-Time PCR system (Life Technologies). The mouse Mapt relative gene expression was evaluated with delta Ct method using Taqman probe sets (Mapt: Mm00521988_m1, GAPDH: 4351309, Life Technologies) and TaqMan Universal PCR Master Mix.

### Brain slice preparation

Immediately following euthanasia via decapitation under deep isoflurane anesthesia, the brain was quickly excised and placed in an ice-cold tissue cutting solution containing (in mM): 194 sucrose, 30 NaCl, 4.5 KCl, 1 MgCl_2_, 26 NaHCO_3_, 1.2 NaH_2_PO_4_, 10 Glucose saturated with a mixture of 95% O2 and 5% CO2, and sliced to a thickness of 280 μm on a vibratome (Leica VT1200S, Germany). Slices were transferred to an artificial cerebrospinal fluid (aCSF) solution containing (in mM): 124 NaCl, 4.5 KCl, 1 MgCl_2_, 26 NaHCO_3_, 1.2 NaH_2_PO_4_, 10 Glucose, 2 CaCl_2_ (310–320 mOsm) saturated with 95% O2/5% CO2 at 30 °C for 1 h before being moved to RT. When ready for recording, in control conditions, slices were transferred to a recording chamber continuously perfused with aCSF solution saturated with 95% O2/5% CO2. In experiments using the ADan oligomer, the slices were first transferred to an incubation chamber containing aCSF containing 400 nM ADan oligomer saturated with 95% O2/5% CO2 at RT for at least 1 h before being transferred to the recording chamber.

### Primary cerebellar granule neurons culture and immunofluorescence

Cerebellums were obtained from 8 to 10 days old mouse pups from WT C57/BL6J (JAX stock #000664) and Tau knock out (JAX stock #007251) mice. Their brains were dissected, the cerebellum separated, cleaned thoroughly from meninges, and incubated with 0.25% trypsin EDTA and 25 mg/mL DNase. Then they were disaggregated by passing them through a 22^1/2^ G syringe needle previously coated with 100 mg/mL BSA in ultrapure cell culture grade water. The suspension was centrifuged for 5 min at 1250 rpm, and then cells were resuspended in 8 mg/mL BSA. A second centrifugation was followed by a resuspension in 2 mg/mL BSA. Finally, cells were resuspended a third time in primary granule neuron media (1X Neurobasal medium, Invitrogen) and counted. They were seeded in 24-well plates that contained coverslips previously treated with 0.01% poly-L-lysine. Seeding number was 50,000 cells/well. After 14 days in vitro (DIV), cells were fixed for 15 min at 37 °C in a solution of 4% paraformaldehyde/sucrose in TBS. Cells were washed with TBS, permeabilized with 0.25% Triton X-100 in TBS, and blocked with 10% BSA in TBS. Primary antibodies anti-Synapsin 1 (1:100, Abcam) and anti-PSD95 (1:100, Abcam) were incubated O.N. at 4 °C. The next day cells were washed in TBS, permeabilized in 0.25% Triton X-100, and incubated with secondary antibodies Alexa Fluor-488 anti-mouse (1:100, Invitrogen) and Alexa Fluor-568 anti-rabbit (1:100, Invitrogen) for 1 h at RT. Finally, cells were washed in TBS for 3 min, mounted with Vectashield mounting medium with DAPI, and visualized using a Leica DMi 8 epifluorescence microscope coupled with the LAS X program (Leica). Clusters of Synapsin 1 and PSD95 were quantified as previously described [14].

### Field potential recordings

Field excitatory postsynaptic potential (fEPSP) recordings from the stratum radiatum of hippocampal area CA1 were carried out at 29–32 °C and aCSF was continuously perfused at a rate of 1–2 ml/minute using a Multiclamp 700B amplifier (Axon Instruments, Union City, CA) signals were amplified (gain 100) and filtered (1 kHz), then digitized (10 kHz). Slices were visualized on an Olympus BX51WI microscope (Olympus Corporation of America). Picrotoxin (50 μM) was added to the aCSF for recordings to isolate excitatory potentials. Micropipettes were prepared from filament-containing borosilicate micropipettes (World Precision Instruments) using a P-1000 micropipette puller (Sutter Instruments, Novato, CA), having a 2.0–3.5 MΩ resistance. The glass micropipettes were filled with 1 M NaCl and placed into the stratum radiatum of hippocampal area CA1. A concentric bipolar stimulating electrode (FHC, Bowdoin, ME) was placed into stratum radiatum in area CA1 ∼ 500 μm from the recording site. fEPSPs were generated by a DS3 Isolated Current Stimulator (Digitimer, Ft. Lauderdale, FL) every 20 s and stimulus intensity was adjusted to produce stable fEPSP responses prior to the initiation of experimental recording. A 10 min baseline was recoded before delivery of four high frequency stimulations (HFS; 100 Hz, 1 s duration, 10 s inter-stimulus-interval). Data were acquired using Clampex 10.3 (Molecular Devices, Sunnyvale, CA). The representative traces were obtained from the average baseline fEPSP (1–10 min) and average post-HFS fEPSP of the final 10 min of recording. Exclusion of individual data points was determined using an outlier calculator included in the Prism 7 software package. Recordings were made 2–7 h after euthanasia. The analysis of fEPSP slopes were used to determine the effects of ADan oligomer. The experimenter was not blinded to treatments administered to the slices.

### Statistical analyses

**Fig. 1:**
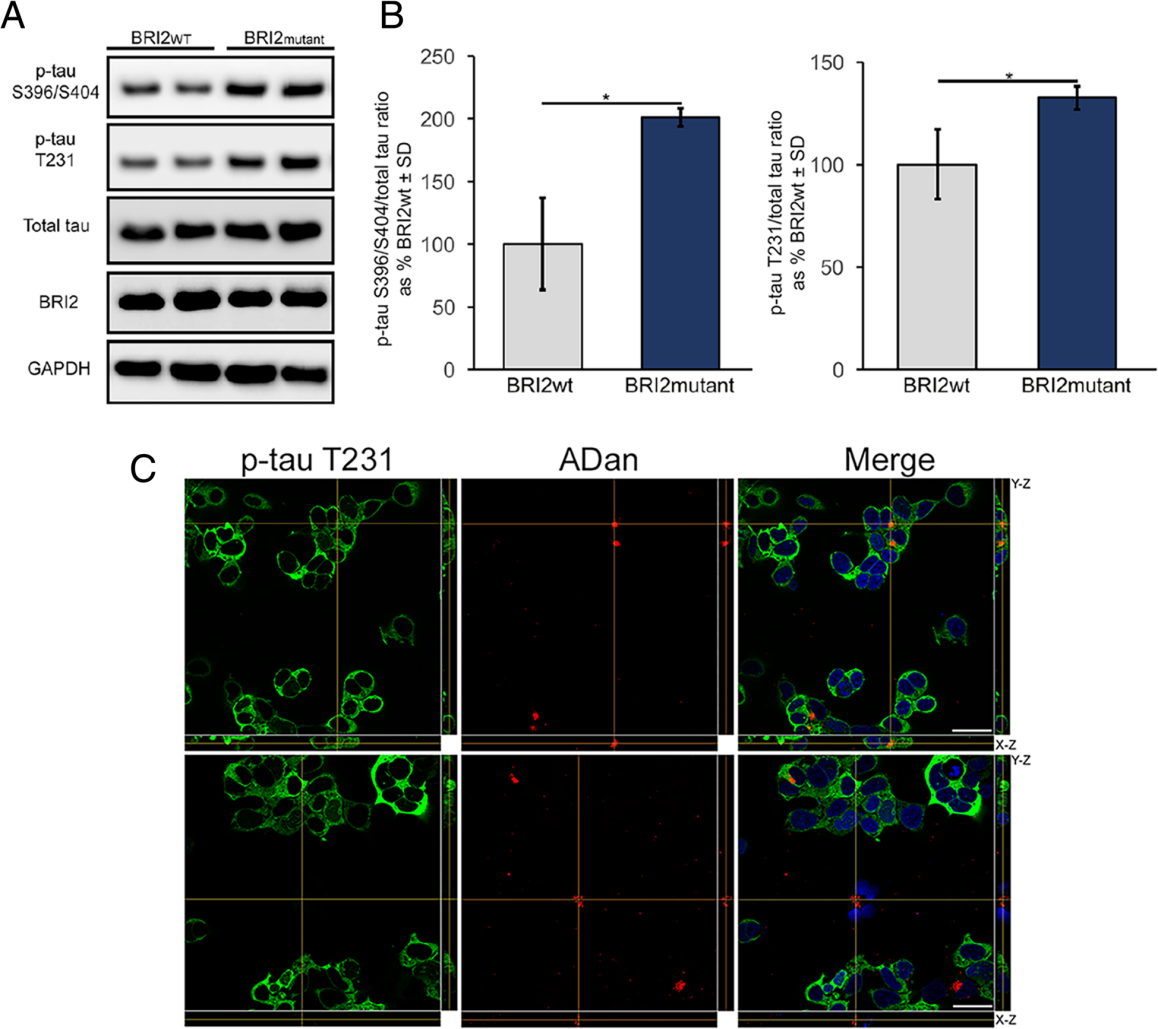
Expression of BRI_2_ bearing the Danish mutation promotes tau phosphorylation. **a** WB analysis of lysates from HEK tau-P301L cells transfected with WT BRI_2_ or mutant BRI_2_. **b** Graph showing WB quantification of p-tau S396/S404/total tau and p-tau T231/total tau ratio. For p-tau S396/S404 analysis; *p = 0.0372, Unpaired Student’s t test. For p-tau T231 analysis; *p = 0.0455, Unpaired Student’s t test. For both quantifications error bars represented ± SD (*n* = 3). Values of phospho-tau/total tau ratio in cells transfected with WT BRI2 were considered as 100%. **c** Orthogonal images of reconstructed three-dimensional views of p-tau T231 (green) and ADan (red) by confocal images of HEK cells expressing human tau-P301L transfected with mutant BRI_2_. Left panels shows staining with anti-p-tau T231 antibody (green), middle panels show staining with anti-ADan antibody (red), and right panels shows the merge of both signals, DAPI staining (blue) for nucleus plus cross-sectional images X-Z and Y-Z. Cross-sectional image in top panel, indicate ADan inclusions that colocalize with p-tau T231. In bottom panel, cross-sectional image indicates ADan inclusions that don’t colocalize with p-tau T231. Scale bar 15 μm

**Fig. 2:**
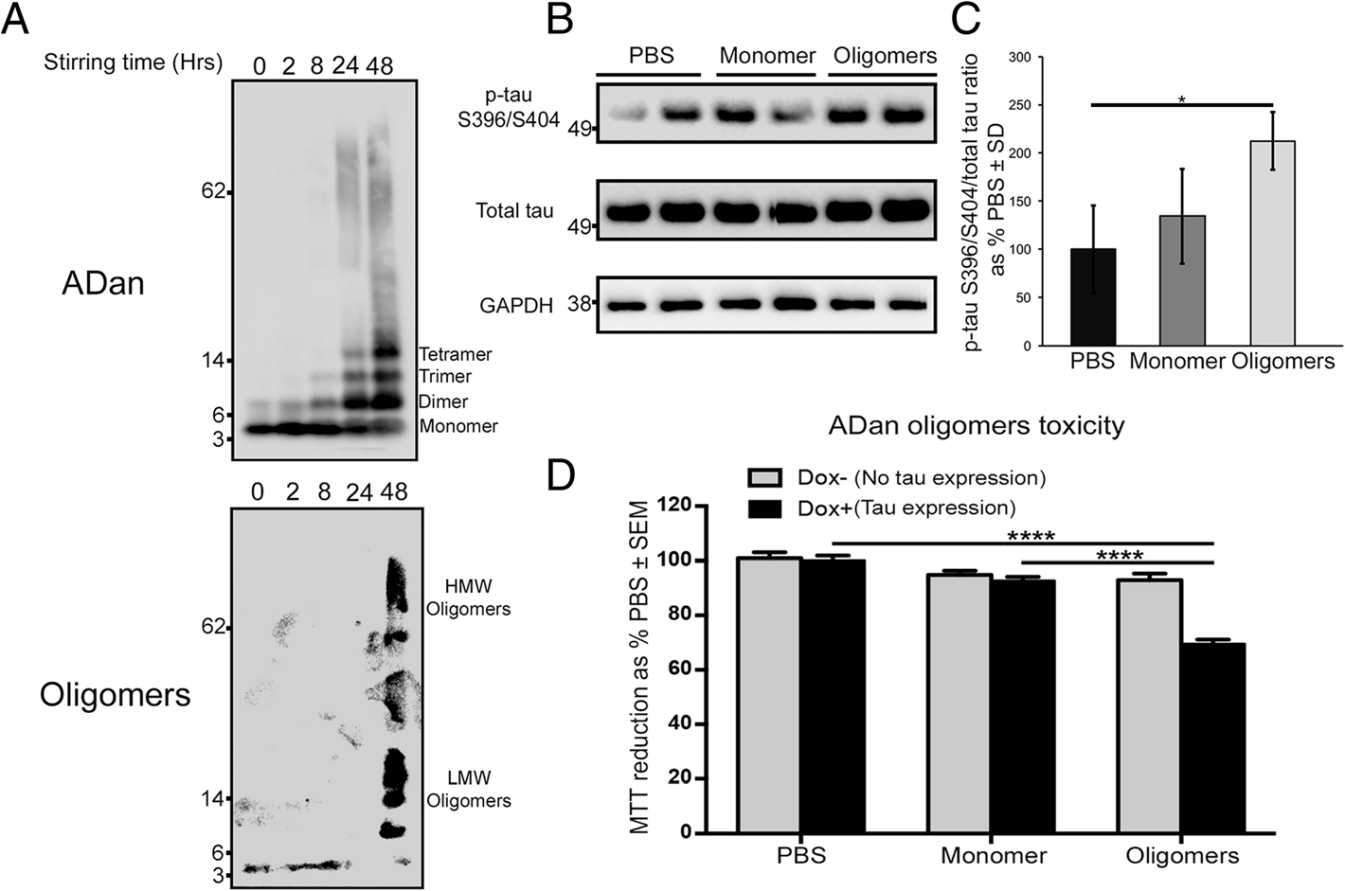
ADan oligomers promoted tau phosphorylation and cellular toxicity that is tau dependent. **a** WB using anti-ADan antibody revealed the formation of multimers from ADan recombinant peptide after 2, 8, 24, and 48 h of stirring. WB using the conformational anti-oligomers antibody F11G3 confirmed the formation of ADan recombinant oligomers after 48 h of stirring. HMW = High Molecular Weight, LMW = Low Molecular Weight. **b** WB analysis of lysates from HEK tau-P301L cells exposed to PBS, ADan monomer or oligomers. **c** Graph showing WB quantification of p-tau S396/S404/ total tau ratio. For p-tau S396/S404 analysis; *p < 0.05, One-way ANOVA with Turkey’s correction. For quantification, error bars represented ± SD (n = 3). Values of phospho-tau S396/S404/total tau ratio in cells treated with PBS were considered as 100%. **d** Cytotoxicity exerted by ADan oligomers in cells, measured by MTT reduction, depends on human tau-P301L expression induced by Dox. For PBS-oligomer analysis in (Dox+); *p < 0.0001, Unpaired Student’s t test. For monomer-oligomer analysis in (Dox+); *p < 0.0001, Unpaired Student’s t test. For quantifications, error bars represented ± SEM (*n* = 6). MTT values were originally obtained as absorbance at 570 nm. For graphic purposes, MTT reduction in cells treated with PBS were considered as 100%

**Fig. 3:**
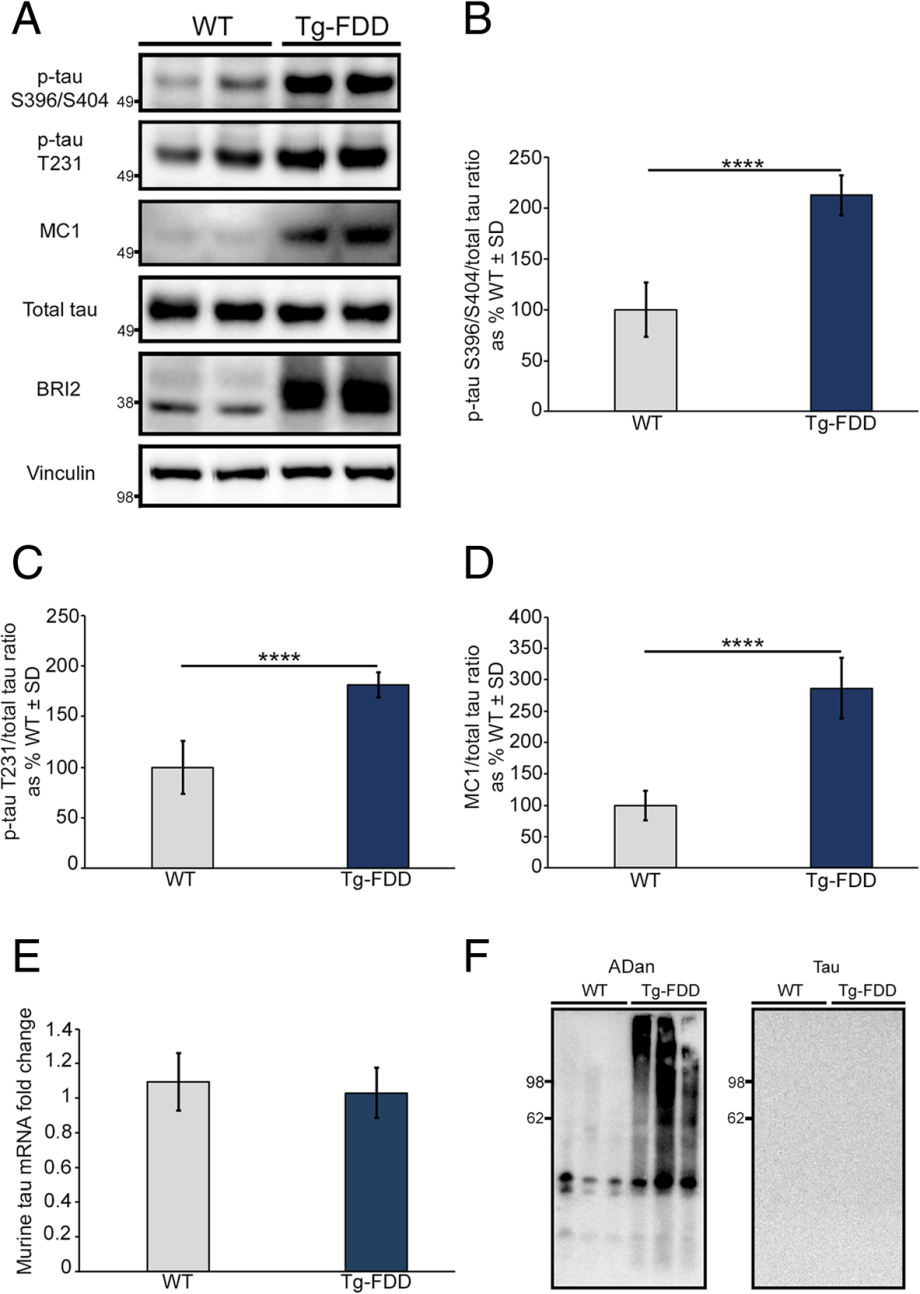
Tau hyperphosphorylation and misfolding in Tg-FDD mice. **a** WB analysis of brain lysate from WT and Tg-FDD 18 months old mice. WB using anti-BRI2 antibody was performed to confirm mice genotypes and Vinculin was used as a loading control. **b** Graph showing WB quantification of p-tau S396/S404/total tau ratio. ****p < 0.0001, Unpaired Student’s t test. **c** Graph showing WB quantification of p-tau T231/total tau ratio. ****p < 0.0001, Unpaired Student’s t test. **d** Graph showing WB quantification of MC1 tau/total tau ratio. ****p < 0.0001, Unpaired Student’s t test. For all three quantifications (**b-d**) error bars represented ± SD (*n* = 5). Values from WT mice were considered as 100%. **(e)** Endogenous murine tau mRNA levels measured by qRT-PCR did not change in Tg-FDD mice in comparison with WT mice (*n* = 5). **f** WB analysis of brain insoluble fractions from WT and Tg-FDD demonstrated the presence of ADan insoluble aggregates in Tg-FDD detected using the anti-ADan antibody (left blot). No tau (using tau-5 antibody) was detected in the insoluble fraction neither from WT nor Tg-FDD mice (right blot)

Experimental analysis and data collection were performed in a blinded fashion, if not stated otherwise. *P* values were determined using the appropriate statistical method via GraphPad Prism, as described throughout the manuscript. Statistical comparisons were made using two-tailed unpaired Student’s t-test with Welch’s correction, Mann-Whitney test or one-Way ANOVA with Tukey’s correction. Figures 1b, 2d, 3b-d, and 8C use unpaired Student’s t-test follow by Welch’s correction. Figure 7b-d use Mann-Whitney test and Fig. 2c use one-Way ANOVA followed by Tukey’s correction. Data are presented as mean ± SD if not stated otherwise. *, **, ***, and **** denote *P* < 0.05, *P* < 0.01, *P* < 0.001, and *P* < 0.0001 respectively. All details of experiments can be found in the Results or the Figure Legends.

## Results

### The ADan peptide leads to hyperphosphorylation of tau

FDD patients are not only characterized by the vascular accumulation of ADan amyloid, but also by the accumulation of hyperphosphorylated tau. Therefore, we decided to determine in a cellular model if ADan promotes tau hyperphosphorylation. To do so, we transfected BRI_2_ bearing the Danish mutation in HEK cells, lacking endogenous tau, that conditionally express human tau with the P301L mutation after the addition of Dox [16]. As a control, we transfected WT BRI_2_. Danish mutant BRI_2_ overexpression led to an increase in the levels of phosphorylated tau at Ser396/Ser404 and Thr231 (Fig. 1a-b). To determine if the effect of ADan aggregates over tau phosphorylation and aggregation is mediated by a direct interaction between ADan peptides and tau or by an indirect mechanism, we performed double-immunostaining using an anti-ADan antibody and an anti-phospho tau antibody (Thr231). We observed colocalization of ADan immunoreactivity with phospho-tau Thr231 in a number of cases, but this was not observed in all cases (Fig. 1c). Overall, these results suggested that the expression of BRI_2_ bearing the Danish mutation could induce the aggregation and phosphorylation of tau, a process that, in cell culture, could be mediated by a direct event or by an indirect mechanism.

### ADan oligomers promote tau phosphorylation and toxicity by a mechanism that is tau dependent

Since BRI_2_ is a transmembrane protein and the Danish mutation induced the secretion of the ADan peptide to the extracellular space [22] (Additional file 1: Figure S2), we evaluated the effect of ADan monomers and oligomers over tau phosphorylation and cellular toxicity. To test this, we prepared ADan recombinant oligomers as we previously described [44]. Figure 2a shows the formation of ADan oligomers immunoreactive with the conformational anti-oligomer F11G3 antibody after 48 h of stirring. Incubation of ADan oligomers or monomers with HEK cells that conditionally express mutant tau for 8 h showed that when cells are exposed to ADan oligomers, tau becomes hyperphosphorylated (Fig. 2b-c). These ADan oligomers and even monomers, in a lower degree, are able to get internalized into the cells (Additional file 1: Figure S3). Moreover, cells treated with oligomers for 4 h showed a significant cellular toxicity as measured by the MTT cell proliferation assay (Fig. 2d). Surprisingly, ADan oligomer toxicity was only observed when cells were induced to express human tau after the addition of Dox, but not when cells did not express it (Fig. 2d). Overall, these results suggest that ADan oligomers could induce tau hyperphosphorylation and subsequent tau-dependent toxicity.

### Accumulation of endogenous murine tau in a mouse model for familial Danish dementia

We tested whether ADan aggregates affect endogenous murine tau phosphorylation in vivo in the Tg-FDD model, which is characterized by ADan amyloid deposits in the vasculature [59] (Additional file 1: Figure S4). WB analysis of soluble brain fractions showed a significant increase in phosphorylated tau at Ser396/Ser404 and Thr231 in 18 months old Tg-FDD mice compared to WT controls of the same ages (Fig. 3a-c). No changes in the levels of phosphorylated tau at Ser202/Thr205, Ser214, Ser262 and Ser356 were observed (data not shown). When we performed WB analysis using the MC1 antibody that recognizes early stages of tau misfolding, we observed a significant increase in MC1-positive tau (Fig. 3a and d), suggesting that the accumulation of vascular amyloid in the Tg-FDD model promotes murine tau misfolding. Quantification of murine tau mRNA levels did not show any differences between Tg-FDD and WT mice (Fig. 3e), demonstrating that the effect of ADan amyloid in the Tg-FDD model over tau is at the protein level. We then performed biochemical characterizations of the insoluble brain fractions from WT and Tg-FDD mice. WB analysis of these fractions demonstrated the presence of insoluble ADan in the Tg-FDD mice (Fig. 3f), but not tau (Fig. 3f) or phospho-tau (data not shown), suggesting that in the Tg-FDD model endogenous murine tau does not accumulate into insoluble aggregates. Interestingly, whenever we performed WB analysis of soluble brain fractions from 3 months old Tg-FDD mice, an age when no vascular amyloid is observed [59], no changes in the level of phosphorylated tau were observed (Additional file 1: Figure S5). Overall, these results suggest that vascular amyloid accumulation possibly induces endogenous tau phosphorylation and misfolding.

**Fig. 4:**
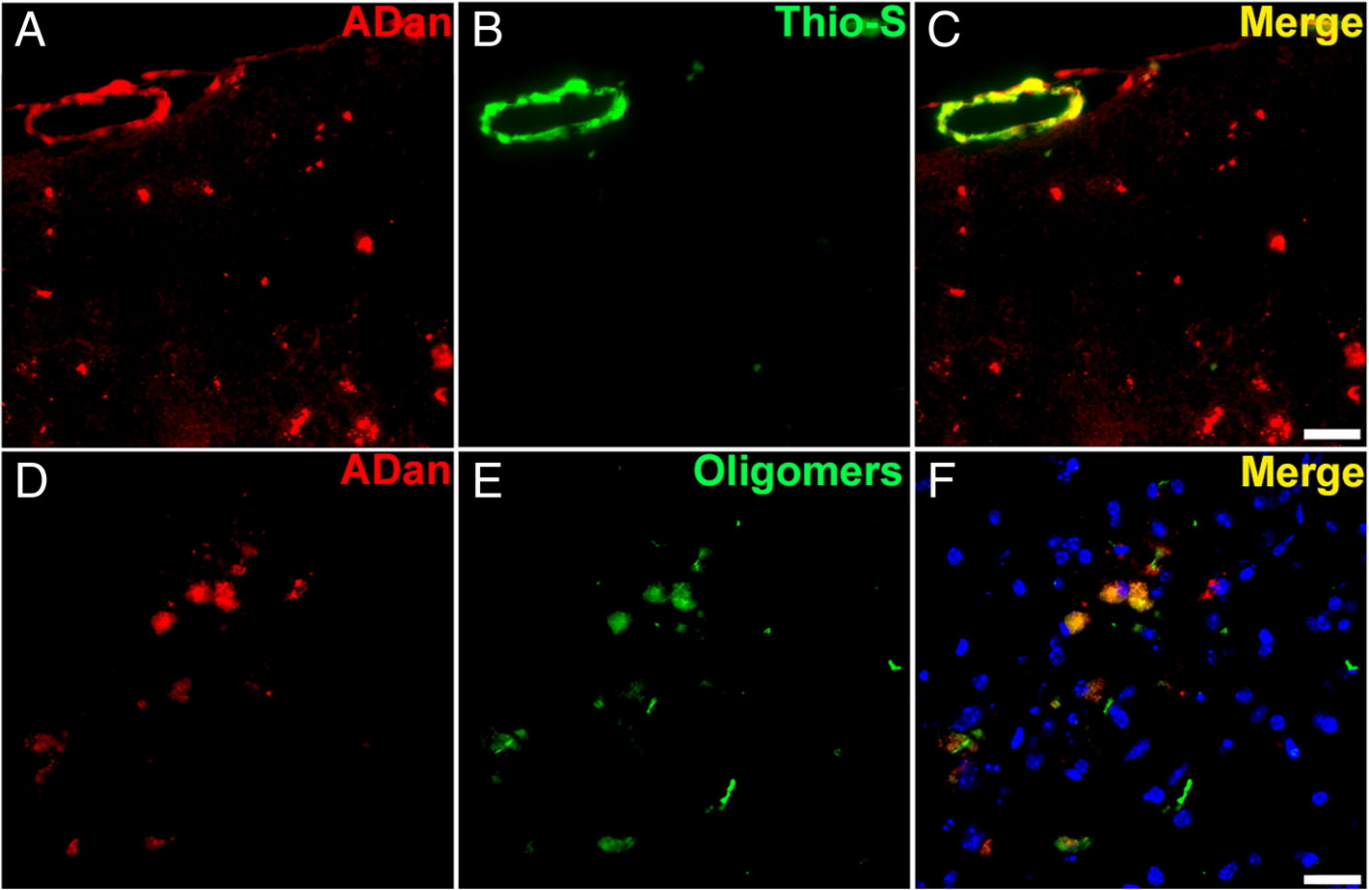
Amyloid characterization in Tg-FDD mice. **a-c** Double immunofluorescence using anti-ADan antibody (**a**, red) and Thio-S (**b**, green) in brain sections of Tg-FDD mice confirm the presence of Thio-S-positive ADan amyloid in the vasculature (**c**, Merge). **e-f** Double immunofluorescence using anti-ADan antibody (**e**, red) and the anti-oligomer antibody F11G3 (**f**, green) in brain sections of Tg-FDD mice confirm the presence of ADan oligomers in the cortex (**f**, Merge). Both are representative images of the cerebral cortex of 18 months old Tg-FD mice. Scale bar 20 μm

To confirm the amyloidogenic nature of ADan deposits in the vasculature of the Tg-FDD model, we double stained brain sections of 18 months old mice with the ADan antibody and Thio-S. We observed that ADan deposition in the cerebral vessels was mainly Thio-S-positive (Fig. 4a-c). Interestingly, small ADan depositions negative for Thio-S were also observed (Fig. 4a-c). To determine the presence of ADan soluble aggregates, such as oligomers, we performed double immunofluorescence in brain sections using the ADan antibody and the conformation specific monoclonal anti-oligomer F11G3 antibody (Fig. 4d-f) [31, 44]. Substantial intracellular co-localization between both antibody signals was observed (Fig. 4f).

**Fig. 5:**
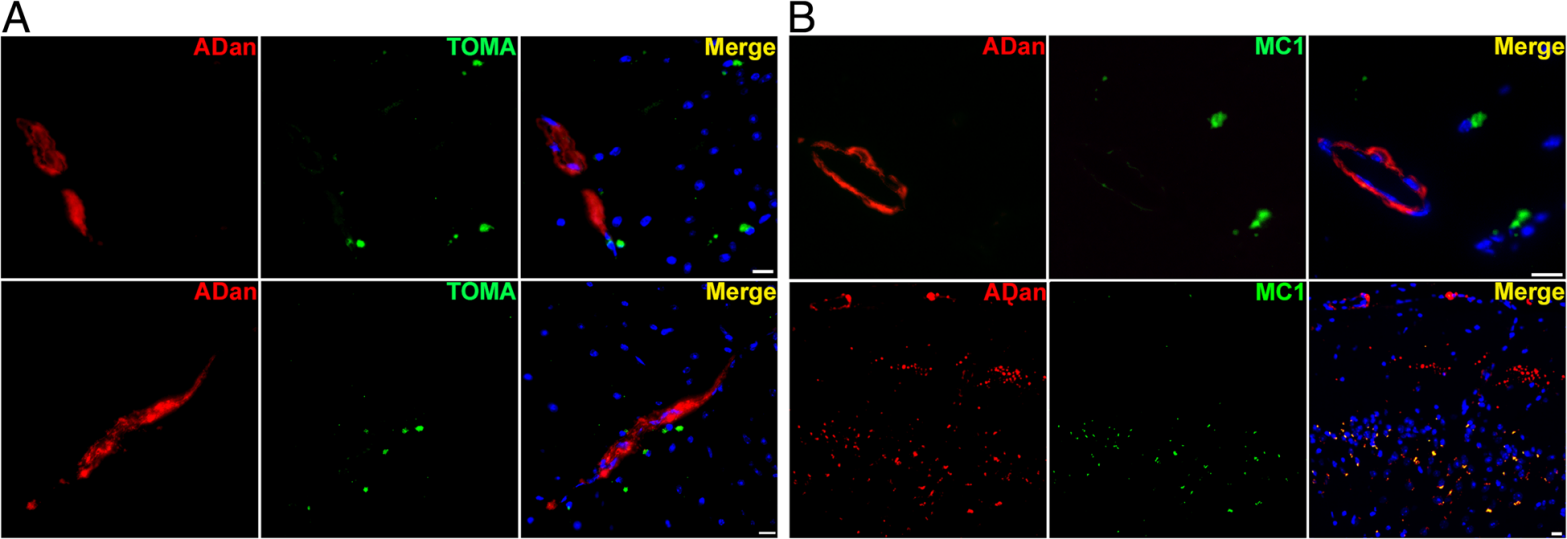
Spatial relationship between tau oligomers and ADan deposits in Tg-FDD mice. **a** Accumulation of tau oligomers detected by TOMA (green) in the vicinity of ADan vascular amyloid detected by anti-ADan antibody (red). **b** Top panel shows accumulation of MC1-positive tau (green) around ADan vascular amyloid (red). Bottom panel shows intracellular localization of ADan deposits (red) and MC1 tau (green) immunoreactivity. All figures were obtained from 18 months old Tg-FDD mice cerebral cortex. Scale bar 15 μm

We performed double immunofluorescence in adjacent sections using anti-ADan and MC1 or TOMA antibodies to assess the spatial relationship between vascular ADan deposits and tau. Both MC1 and TOMA antibodies recognized early stages of tau aggregation such as oligomers [11, 37]. Double staining of anti-ADan and TOMA on Tg-FDD sections showed the accumulation of tau oligomers in the vicinity of vessels enriched with amyloid deposits in the cortex (Fig. 5a, top and bottom panel). Double staining of anti-ADan and MC1 confirmed the presence of misfolded tau surrounding ADan-positive vessels (Fig. 5b, top panel). Interestingly, double staining of anti-ADan and MC1 also revealed intracellular association between ADan and MC1-positive tau in the cerebral cortex (Fig. 5b, bottom panel). Overall these results demonstrated the presence of two types of pathological accumulation of tau in this CAA model. One where misfolded tau accumulates in the vicinity of vessels enriched with ADan deposits, and a second one where misfolded tau co-localized with ADan intracellular signal in areas of the cerebral cortex, where no major vascular amyloid deposits are observed. Tau oligomers were observed in other brain regions (Additional file 1: Figure S6) that have been previous associated with the accumulation of vascular amyloid in this mouse model [59].

### Tau oligomeric deposits in the vicinity of vascular amyloid are closely associated with astrocytic profile

**Fig. 6:**
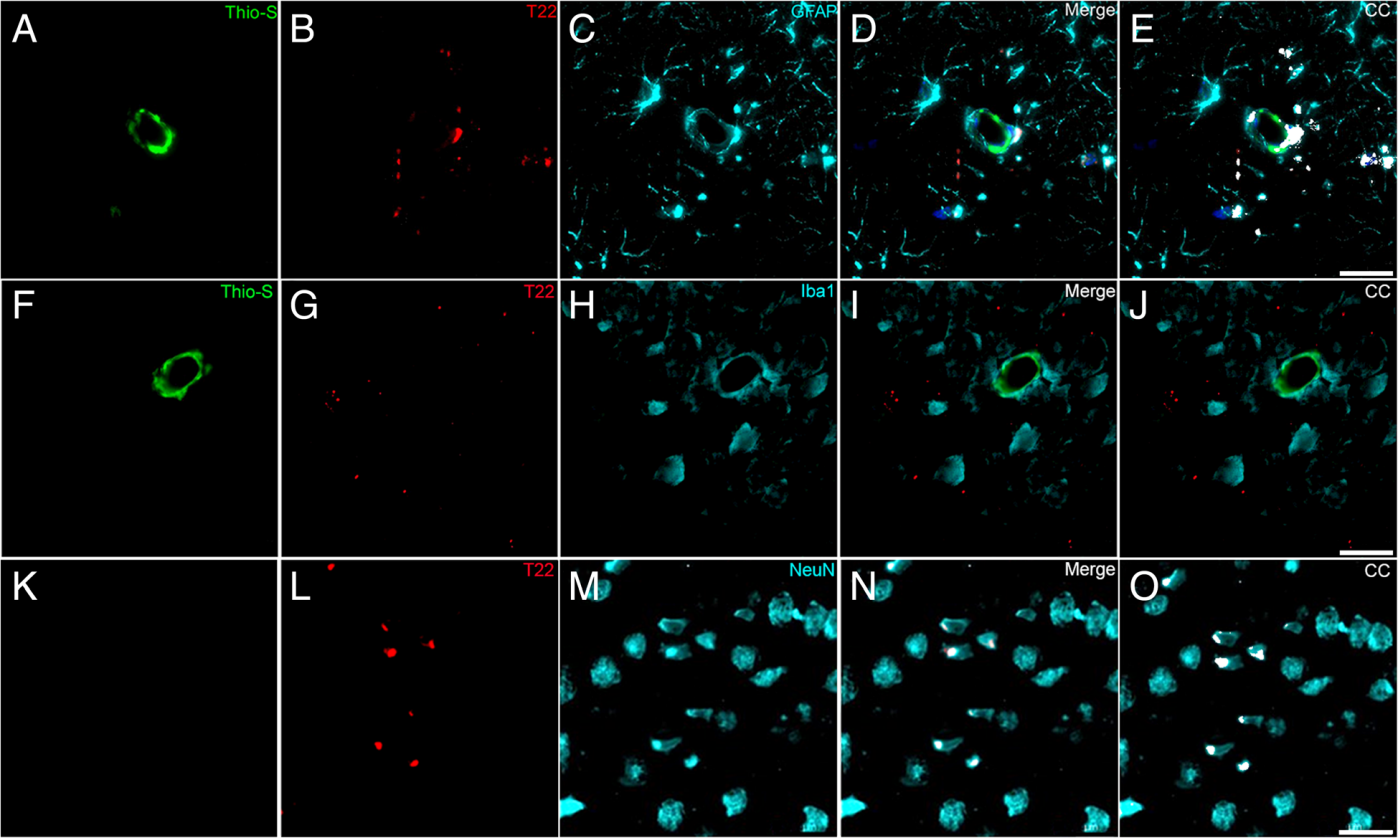
Tau oligomers cell specificity in Tg-FDD mice. **a-e** Fluorescent microscopic images of Tg-FDD brain section triple immunostaining for amyloid (Thio-S, green), Tau oligomers (T22, red), and astrocytes (GFAP, cyan). Tau oligomers immunoreactivity showed close association with astrocytes and astrocytic processes (Merge). Colocalization analysis (CC) was performed to determine pixel intensity correlation between T22 and GFAP signals. White pixels indicate colocalization between T22 and GFAP signal (**e**, white pixels). **f-j** Triple immunostaining for amyloid (Thio-S, green), Tau oligomers (T22, red), and microglia (Iba1, cyan). Tau oligomers are not associated to microglia surrounding vascular amyloid (Merge). Colocalization analysis confirmed the lack of correlation between T22 and Iba1 signal (**j**). **k-o** Triple immunostaining for amyloid (Thio-S, green), Tau oligomers (T22, red), and neurons (NeuN, cyan). Tau oligomers were found associated to neurons in areas where vascular amyloid was not detected. White pixels indicate colocalization between T22 and NeuN signal (**o**, white pixels). All figures were obtained from 18 months old Tg-FDD mice cerebral cortex. Scale bar 25 μm

Innate immunity is activated both in cerebrovascular disease [38] and in AD. In the context of our CAA model, we observed severe astro and microgliosis, as evidenced by robust GFAP and Iba1 staining in areas affected by ADan amyloid vascular deposits (Additional file 1: Figure S7). Based on this observation, we decided to determine which cell type showed tau oligomers associated with vascular amyloid. We performed triple immunofluorescence in Tg-FDD brain sections for Thio-S (amyloid), T22 (tau oligomers), and GFAP or Iba1 or NeuN for astrocytes, microglia, or neurons, respectively. Fluorescent microscopy suggests that tau oligomers are closely associated with GFAP-positive astrocytes (Fig. 6a-e). Co-localization analysis between T22 and GFAP signal suggest that these oligomers are located in the cytoplasm and astrocytic processes (Fig. 6e). Triple staining with Thio-S, T22, and Iba1 showed that tau oligomers were not associated with Iba1-positive microglia (Fig. 6f-j). Interestingly, triple staining revealed that tau oligomers are associated with neurons in areas throughout the cortex where no Thio-S-positive vascular structures were located (Fig. 6k-o). Substantial co-localization between T22 and NeuN antibody signals was observed (Fig. 6o).

### Tau is required for ADan-induced impairment of the structure and function of neuronal synapses

It is widely accepted that the adverse effects of Aβ on neuronal degeneration and cognitive dysfunction appear to depend largely on soluble tau [29]. For instance, it has been reported that the reduction of tau expression prevents Aβ-induced neurodegeneration in cell culture [51, 65]. Moreover, in vivo studies have shown how genetic ablation of endogenous murine tau in a hAβPP mouse model prevents behavioral deficits without altering the amount of Aβ plaques in the parenchyma [54]. Therefore, considering these previous studies, in addition to the fact that a decrease in the levels of synaptic markers in Tg-FDD mice has been reported [23], we aimed to investigate if the neuronal damage triggered by ADan, a highly vasculotropic amyloid, is also dependent on tau.

**Fig. 7:**
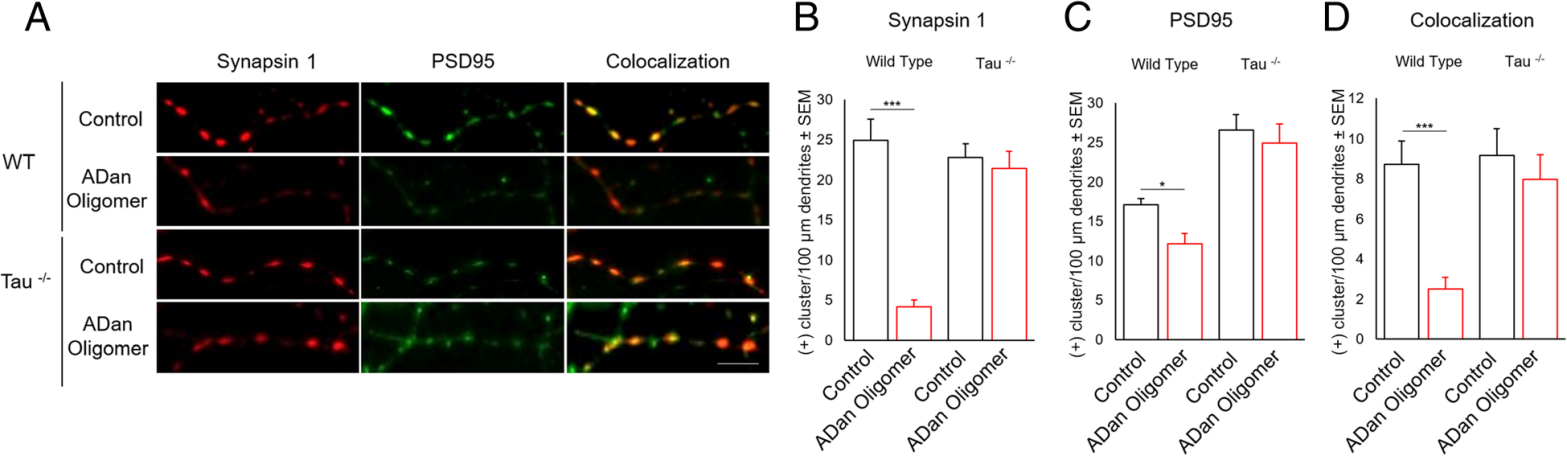
ADan oligomers do not alter the number and localization of synaptic proteins in Tau^−/−^ mice derived cerebellar granule neurons. **a** Representative immunofluorescence photographs for clusters of the synaptic proteins Synapsin-1 (red), PSD95 (green), and their colocalization (yellow) in cerebellar granule neuronal dendrites. Four experimental groups were analyzed: WT and Tau^−/−^ neurons in the presence or absence of ADan oligomers (+ADan, **b-d**) Graphs showing the quantification of positive ((+)) clusters for each marker and their colocalization per 100 μm of dendrite. The number of cells analyzed was 15 per each culture, from 3 independent cultures. Results are shown as the mean ± SEM of *n* = 3. Asterisks indicate significant differences, where * p < 0.05 and *** *p* < 0.001 by Mann-Whitney test. Scale bar = 10 μm

**Fig. 8:**
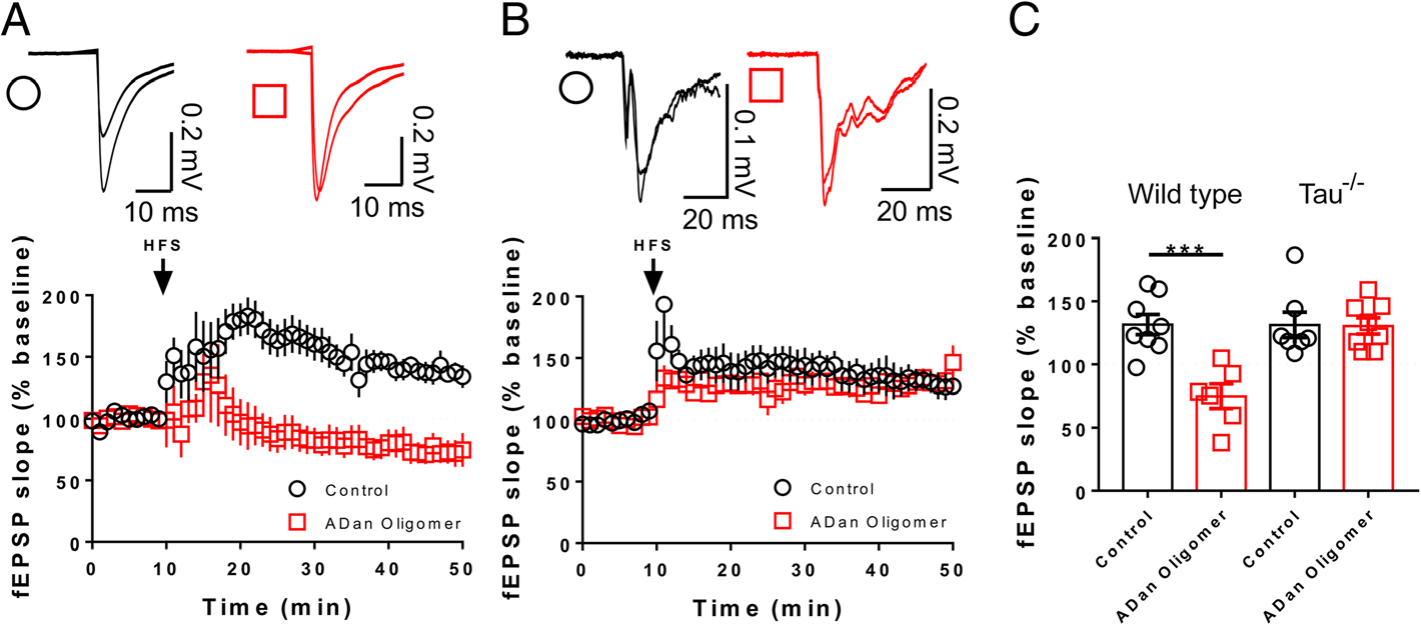
ADan oligomers do not reduce LTP in slices of Tau^−/−^ mice. **a** ADan oligomers reduce LTP in slices of WT mice. Hippocampal Schaffer collateral-CA1 LTP in WT mice in control (circles) or after incubation with ADan oligomers (squares). Representative traces are the average baseline fEPSP (1–10 min) and average fEPSP of the final 10 min of recording (40–50 min) for each condition. **b** Hippocampal Schaffer collateral-CA1 LTP in Tau^−/−^ mice in control (circles) or after incubation with ADan oligomers (squares). ADan oligomers do not reduce LTP in slices of Tau^−/−^ mice. The inset shows superimposed example traces before and 40 min after high-frequency stimulation for each condition. **c** Data show average of normalized fEPSP slope for final 10 min of recording (40–50 min) relative to 10 min baseline average. For WT; *p* = 0.0007, t_12_ = 4.537, Unpaired Student’s t test; *n* = 8 slices Ctrl from 4 mice, n = 6 slices ADan oligomer (400 nM) from 4 mice. For Tau^−/−^; *p* = 0.9440, t_13_ = 0.0716, Unpaired Student’s t test; *n* = 7 slices Ctrl from 4 mice, n = 8 slices ADan oligomer (400 nM) from 4 mice

To investigate if ADan aggregates induce impairment of the synaptic structure via tau, we performed IF for the pre- and post-synaptic markers Synapsin-1 and PSD95 respectively, and assessed their localization at synapses in cultured primary cerebellar granule neurons from WT mice and mice with a genetic knock-out of *Mapt* gene (Tau^−/−^) in the presence or absence of ADan oligomers (Fig. 7a). Our results show a decrease in the total number of clusters or puncta marked for Synapsin-1 and PSD95 in WT neurons incubated with 500 nM of ADan oligomers in comparison with untreated WT control groups. Remarkably, ADan oligomers did not have any effect on the number of clusters marked either for Synapsin-1 or PSD95 on Tau^−/−^ neurons (Fig. 7b-c). ADan oligomers not only affected the number of Synapsin-1 and PSD95 clusters in WT neurons, but also has a detrimental effect on the colocalization between these pre- and post-synaptic markers. On the other hand, no effect on the synaptic colocalization between Synapsin-1 and PSD-95 was observed in Tau^−/−^ neurons after the addition of ADan oligomers (Fig. 7d). To correlate these results with a functional analysis, we performed hippocampal long-term potentiation (LTP) experiments in hippocampal area CA1 in WT and Tau^−/−^ mouse-derived brain slice preparations. We monitored fEPSPs evoked by extracellular stimulation of the Schaffer collateral pathway and induced LTP using high-frequency stimulation (four stimuli of 100 Hz for 1 s with a 10 s inter-stimulus interval). In WT slices pretreated with 400 nM ADan oligomers for 2 h, LTP was almost completely blocked (Fig. 8a and c). This was a specific effect of ADan oligomers, because control slices showed LTP (Fig. 8a and c). These results demonstrate that, in WT mice, acute application of ADan impairs one or more of the cellular mechanisms necessary for LTP. To confirm a possible functional interaction between ADan and tau, we tested the effect of ADan oligomers on LTP in Tau^−/−^ mice. Slices incubated in the control solution showed normal levels of LTP and, remarkably, slices preincubated in ADan oligomers showed LTP levels of similar magnitude (Fig. 8b-c).

Overall, these results indicate that the absence of tau could prevent the synaptic dysfunction induced by a CAA-associated amyloid such as ADan.

## Discussion

The data reported here shows that ADan amyloid peptide, the amyloid responsible for wide spread CAA in FDD patients, induces the phosphorylation and misfolding of tau in vitro and in vivo. We also demonstrated that ADan oligomers exert cellular toxicity and hippocampal LTP impairment in brain slices in a tau dependent manner.

Previous studies reported the existence of several neurodegenerative diseases characterized by vascular amyloid deposition known as CAA [52, 58]. Among CAAs, Aβ-CAA is the most common and is frequently present in AD [42]. Other examples include the accumulation of vascular ADan amyloid or ABri amyloid in patients with Familial Danish Dementia and Familial British Dementia, respectively [61, 63]. Vascular accumulation of amyloidogenic forms of Cystatin C, Gelsolin, Transthyretin, and prion protein are pathological hallmarks of Hereditary cystatin C amyloid angiopathy, Familial amyloidosis Finnish type, Familial transthyretin amyloidosis (FTA)-leptomeningeal form and prion protein-CAA, respectively [18, 24, 45, 52, 62]. Interestingly, in some forms of CAA, glial activation and aggregated tau deposits are present, suggesting an important role for inflammation and tau accumulation in neurodegeneration associated with vascular amyloid deposition, as has been previously suggested for parenchymal amyloid accumulation [8, 57].

Noteworthy, several studies have suggested that pathologic changes of tau in neurons can impact brain endothelial cell biology, altering the integrity of the brain’s microvasculature [5, 47]. For instance, it has been shown that vessel wall remodeling of leptomeningeal arteries is an early-onset, tau pathology-dependent process, which may potentially contribute to downstream CAA-dependent microvascular pathology in AD patients [47]. Even more, other studies have demonstrated an increase of BBB permeability and the accumulation of tau oligomers in the cerebral microvasculature of human patients with progressive supranuclear palsy (PSP) [4, 10], emphasizing the role of tau aggregates in the functional and structural integrity of the cerebral vasculature. In a similar fashion, tau-overexpressing mice develop changes to blood vessels including abnormal, spiraling morphologies; reduced blood vessel diameter; and increased overall blood vessel density in the cortex [5]. Also, in a different mouse model for tauopathies, BBB dysfunction emerges at the same time that perivascular tau emerges around major hippocampal blood vessels. However, when tau expression is suppressed, BBB integrity is preserved, suggesting that the BBB can be stabilized in a tauopathic brain by reducing tau levels [7]. Overall, these studies strongly suggest that in relation to vascular damage, tau aggregation is not a unidirectional event where vascular damage initiates a series of events that triggers tau aggregation, but is rather a vicious cycle where tau pathology enhances vascular damage.

In Tg-FDD mice, expression of the Danish mutant form of BRI_2_ driven by the moPrnp promoter is sufficient for the development of CAA, suggesting that neural cells are the source of the cerebrovascular amyloid and highlighting the vasculotropic nature of the ADan peptide [59]. In the present study, we confirmed the presence of a robust activation of micro and astroglia surrounding vascular ADan deposits in Tg-FDD mice. We biochemically demonstrated an increase of hyperphosphorylated forms of tau in the Tg-FDD. This increase was only observed in certain phospho-tau epitopes, but not others that have been associated to tau NFTs in AD, suggesting that the process of tau hyperphosphorylation associated to CAA deposits could be different from the hyperphosphorylation of tau associated to parenchymal deposition of amyloid. We also showed the presence of tau oligomers in perivascular regions, in the vicinity of ADan amyloid deposits, as well as in areas throughout the cerebral cortex. Based on our observations, at least in the context of FDD, we proposed a dual-mechanism of action with vascular amyloid deposits resulting in tau hyperphosphorylation/aggregation and subsequent neurodegeneration:

1. Indirect mechanism is where ADan peptides secreted from neurons accumulate around vascular vessels. Subsequently, vascular amyloid accumulation triggers perivascular astrogliosis and astrocytic tau oligomerization. Interestingly, astrocytes play a key role in maintaining the BBB via astrocytic end feet directly opposed to vascular endothelial cells [50], and tau has been shown to accumulate in these end feet in tauopathies [36, 41], including perivascular astrocytic tau deposits in CAA patients [60]. These results suggest that vascular damage, due to amyloid accumulation, could induce an astrocytic response that triggers tau misfolding.

In addition to maintaining BBB integrity, astrocytes also modulate synaptic function by the secretion and uptake of neurotransmitters such as glutamate [32]. Considering this, plus a recent description of a frontotemporal dementia variant that displayed reduced glutamate transporter-1 staining in a subset of tau-bearing astrocytes [21], and also that the sole presence of tau in astrocytes in a mouse model for tauopathies produces a significant decrease in glutamate transport capacity [17]; we can speculate that the decrease in synaptic markers observed in the Tg-FDD mouse model could be due to astrocytic dysfunction triggered by tau oligomers. Nevertheless, considering novel studies where the role of glia in relation to tau spreading is described [2, 46], we cannot rule out the propagation of tau oligomers from the astrocytes to neurons as a source of synaptic function disturbance in the Tg-FDD model.

2) Direct mechanism is where ADan peptide, cleaved from transmembrane neuronal BRI_2_, forms amyloid oligomers and is internalized by neurons. These intraneuronal aggregates could directly induce the aggregation of endogenous tau by a cross-seeding event that subsequently promotes synaptic dysfunction. A similar mechanism has been suggested for Aβ and alpha-synuclein, in which both amyloids interact with tau via an interface to co-aggregate into hybrid oligomers [31, 56]. We cannot exclude the possibility that the intraneuronal ADan could co-aggregate with tau in the Tg-FDD due to the neuronal over-expression of mutant BRI_2_ rather than a CAA specific phenotype. Nevertheless, it has been previously reported that Aβ_40_, an amyloid highly associated with Aβ-CAA, can also form intraneuronal inclusions [30], suggesting that CAA-associated amyloids could also contribute to neurodegeneration via the formation of neuronal aggregates.

Overall, both proposed mechanisms enhance the relevance of tau in the pathogenesis associated to CAA. This is highly relevant considering a novel patient-based study where it was shown that tau burden could play a pivotal role in cognitive impairment in patients with subcortical vascular cognitive impairment (SCVI), suggesting that tau could represent a common pathway for dementia triggered by both cerebrovascular and parenchymal amyloid pathologies [40].

Previous studies have shown how endogenous WT tau appears to be required for parenchymal Aβ-amyloid accumulation and ApoE4 to cause synaptic, network, and cognitive deficits in mouse models of AD [1, 53, 54]. Tau ablation has also been shown to attenuate motor abnormalities in a Huntington’s disease (HD) mouse model [20] and prevent deficits in spatial learning and memory after repeated mild frontal impact in WT mice [13]. Even more, tau reduction has the ability to block epileptogenesis of diverse causes, including epileptic activity triggered by pharmacological blockade of GABA_A_ channels [19, 54], genetic ablation of the voltage-gated potassium channel subunit Kv1.1 [33], depletion of ethanolamine kinase or of the K^+^–Cl^−^ cotransporter [33], or depletion of the voltage-gated sodium channel subunit Nav1.1 [26]. The mechanisms underlying these beneficial effects of tau reduction remain to be determined [48]. As mentioned above, recent evidence suggest that tau could be involved in a common pathway for neurodegeneration triggered by cerebrovascular abnormalities and parenchymal amyloid pathologies [40]. Thus, our findings that demonstrated how ADan amyloid aggregates required endogenous tau expression to exert cell death; synaptic and LTP impairment lead us to suggest that partial tau reduction could be considered a feasible approach for the treatment of dementias associated with CAA, at least in the context of FDD.

## Conclusions

Previous efforts in AD and AD-related dementias have aimed to understand the connection between parenchymal amyloid, tau aggregation, and neurodegeneration, with the contribution of vascular amyloid pathology to tau aggregation and neurodegeneration remaining understudied. To the best of our knowledge, this is the first study aiming to understand, in detail, the connection between vascular amyloid deposition and tau pathology. Using a set of in vitro and in vivo approaches, we proposed the existence of two possible mechanisms of how ADan vascular amyloid may trigger tau misfolding. Even more, the fact that tau reduction was sufficient to prevent neuronal synaptotoxicity due to the presence of ADan oligomers, an amyloid highly associated to vascular deposits substantiates, at least in FDD, tau level modulation as an effective therapeutic target for neurodegeneration associated with CAA.

## Additional file

Additional file 1: **Figure S1**. Doxycycline-inducible cell line expressing human mutant P301L tau. Western blot of cell lysate probed with Tau-5 antibody confirming the expression of tau induced by Dox. **Figure S2**. BRI2 processing in Familial Danish Dementia. Processing of mutant BRI2 by pro-protein convertases (PCs) generates the 34 amino acid peptide (ADan) and a mature form of BRI2 (m-BRI2). Processing by ADAM10 in the ectodomain of BRI2 releases the BRICHOS domain and an N-terminal fragment. Disulfide bonded loops in the BRICHOS domain and in the carboxy-terminus of BRI2 are indicated. **Figure S3**. ADan oligomers and monomers are internalized in HEK cells. Orthogonal images of reconstructed three-dimensional views of ADan (red) and nucleus (blue) by confocal images of HEK tau-P301L cells. ADan oligomers are internalized and accumulated intracellularly (right image). In a lower degree than oligomers, ADan monomers are internalized into cells too (middle image). PBS treated cells were utilized as negative controls. **Figure S4**. CAA in a transgenic mouse model for FDD: Thio-S detection of leptomeningeal and cortical blood vessels in the cerebellum and cortex of Tg-FDD mice. **Figure S5**. Young Tg-FDD mice do not show changes in tau. **(A)** Western blot of brain from 3 months old WT and Tg-FDD mice. **(B)** Graph showing WB quantification of p-tau S396/S404. **Figure S6**. Tau oligomers in Tg-FDD mice. IF using the TOMA antibody (green) revealed the presence of tau oligomers in the hippocampus, cortex, and cerebellum of 18 months old Tg-FDD mice. MC1-positive staining was also observed in the hippocampus, cortex, and cerebellum of these mice. Tau-/- was utilized as control. **Figure S7**. Glial activation associated to CAA. **(A-F)** IF of ADan amyloid (red) and GFAP (green) in Tg-FDD **(A-C)** and WT **(D-F)**. **(G-L)** IF of ADan amyloid (red) and Iba1 (green) in Tg-FDD **(G-I)** and WT **(J-L)**. Scale bar 25 μm. (DOCX 10546 kb)

## Abbreviations

ABri: British amyloid
AD: Alzheimer’s disease
ADan: Danish amyloid
ADRD: Alzheimer’s disease related dementias
BBB: Blood brain barrier
CAA: Cerebral amyloid angiopathy
DIV: Days in vitro
Dox: Doxycycline
FA: Formic acid
FBD: Familial British Dementia
FDD: Familial Danish Dementia
fEPSP: Field excitatory postsynaptic potential
IHC: Immunohistochemistry
LTP: Long term potentiation
Mapt: Microtubule associated protein tau
NFTs: Neurofibrillary tangles
PSP: Progressive supranuclear palsy
RT: Room temperature
Thio-S: Thioflavine S
WB: Western blot
