# Supplementary material for: Tau as a mediator of neurotoxicity associated to cerebral amyloid angiopathy
Source: Acta Neuropathol Commun. 2019 Feb 26;7:26. doi: 10.1186/s40478-019-0680-z (PMC6390363; doi:10.1186/s40478-019-0680-z)
Supplement: Supplementary file 1 — Figure S1. Doxycycline-inducible cell line expressing human mutant P301L tau. Western blot of cell lysate probed with Tau-5 antibody confirming the expression of tau induced by Dox. Figure S2. BRI2 processing in Familial Danish Dementia. Processing of mutant BRI2 by pro-protein convertases (PCs) generates the 34 amino acid peptide (ADan) and a mature form of BRI2 (m-BRI2). Processing by ADAM10 in the ectodomain of BRI2 releases the BRICHOS domain and an N-terminal fragment. Disulfide bonded loops in the BRICHOS domain and in the carboxy-terminus of BRI2 are indicated. Figure S3. ADan oligomers and monomers are internalized in HEK cells. Orthogonal images of reconstructed three-dimensional views of ADan (red) and nucleus (blue) by confocal images of HEK tau-P301L cells. ADan oligomers are internalized and accumulated intracellularly (right image). In a lower degree than oligomers, ADan monomers are internalized into cells too (middle image). PBS treated cells were utilized as negative controls. Figure S4. CAA in a transgenic mouse model for FDD: Thio-S detection of leptomeningeal and cortical blood vessels in the cerebellum and cortex of Tg-FDD mice. Figure S5. Young Tg-FDD mice do not show changes in tau. (A) Western blot of brain from 3 months old WT and Tg-FDD mice. (B) Graph showing WB quantification of p-tau S396/S404. Figure S6. Tau oligomers in Tg-FDD mice. IF using the TOMA antibody (green) revealed the presence of tau oligomers in the hippocampus, cortex, and cerebellum of 18 months old Tg-FDD mice. MC1-positive staining was also observed in the hippocampus, cortex, and cerebellum of these mice. Tau-/- was utilized as control. Figure S7. Glial activation associated to CAA. (A-F) IF of ADan amyloid (red) and GFAP (green) in Tg-FDD (A-C) and WT (D-F). (G-L) IF of ADan amyloid (red) and Iba1 (green) in Tg-FDD (G-I) and WT (J-L). Scale bar 25 μm. (DOCX 10546 kb) [file 40478_2019_680_MOESM1_ESM.docx]

**Additional file 1: Supplementary Figures**


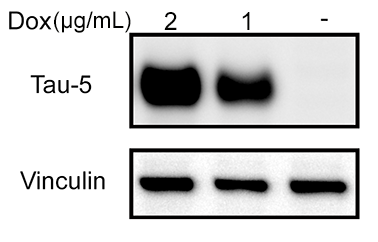


**Figure S1: Doxycycline-inducible cell line expressing human mutant P301L tau.** Western blot of cell lysate probed with Tau-5 antibody confirming the expression of tau induced by doxycycline (Dox). Vinculin was used as a loading control.


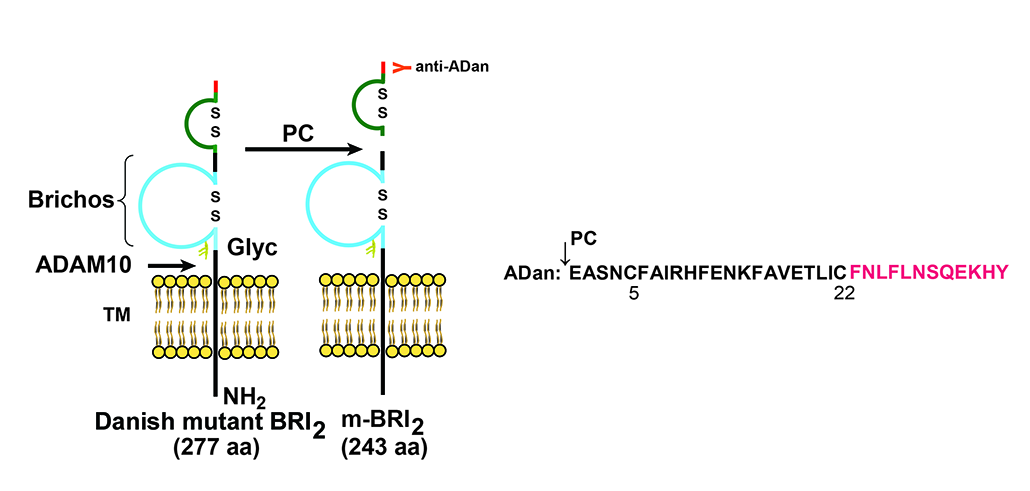


**Figure S2: BRI_2_ expression and processing in Familial Danish Dementia.** Schematic diagram of the Danish mutant form of BRI_2_. BRI_2_ is a type-II single trans-membrane (TM) domain protein. Processing of mutant BRI_2_ by pro-protein convertases (PCs) generates the 34 amino acid peptide (ADan) and a mature form of BRI_2_ (m-BRI_2_). Processing by ADAM10 in the ectodomain of BRI_2_ releases the BRICHOS domain and an N-terminal fragment (NTF). The NTF is also the subject of additional proteolysis by SPPL2, releasing an intracellular domain (ICD) and a C-terminal peptide fragment (BRI_2_ C-peptide). Disulfide bonded loops in the BRICHOS domain and in the carboxy-terminus of BRI_2_ (amino acids 5 and 22 of the ADan peptide) are indicated. The figure shows the Abs targeted epitopes.

**Figure S3: Recombinant ADan oligomers and monomers are internalized in HEK cells.** Orthogonal images of reconstructed three-dimensional views of ADan (red) and nucleus (DAPI, blue) by confocal images of HEK tau-P301L cells. Representative images demonstrated that ADan oligomers are internalized and accumulated intracellularly (right image). In a lower degree than oligomers, ADan monomers are internalized into cells too (middle image). PBS treated cells were utilized as negative controls (left image). Cross-sectional images X-Z and Y-Z were used to determine ADan oligomers or monomers internalization given by the presence of DAPI signal (nucleus) in the same focal plane.


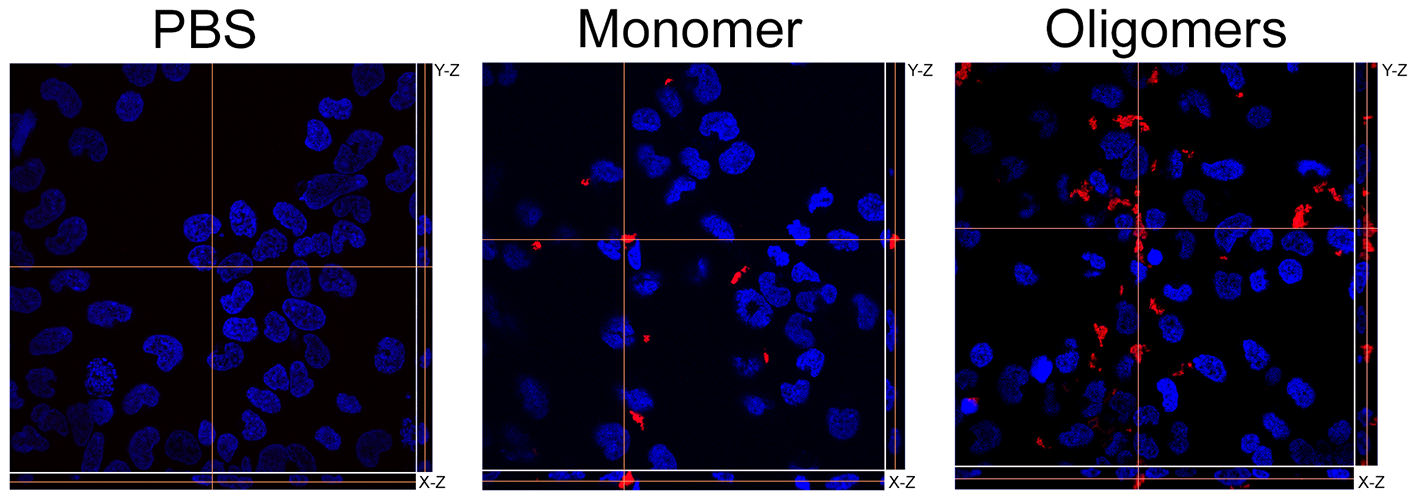


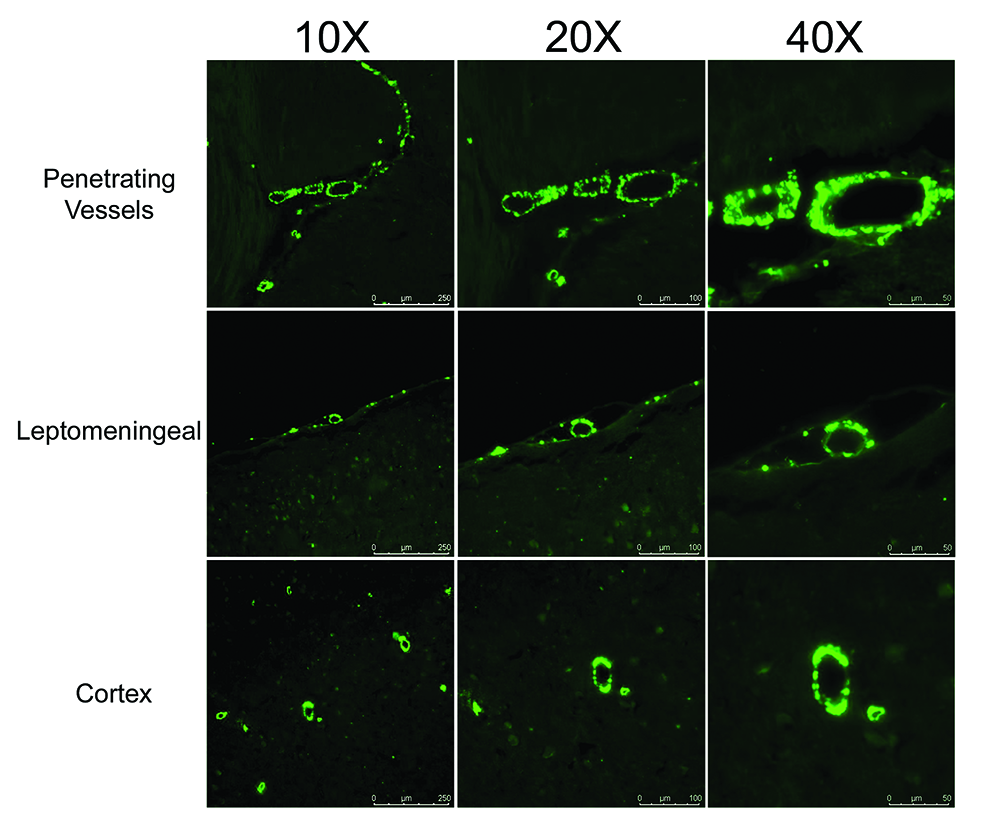


**Figure S4: CAA in a transgenic mouse model for Familial Danish Dementia mice:** Vascular amyloid deposition is observed in leptomeningeal and penetrating vessels in the cerebellum and cortex of transgenic-Familial Danish Dementia (Tg-FDD) mice. Thio-S detection of leptomeningeal and cortical blood vessels.


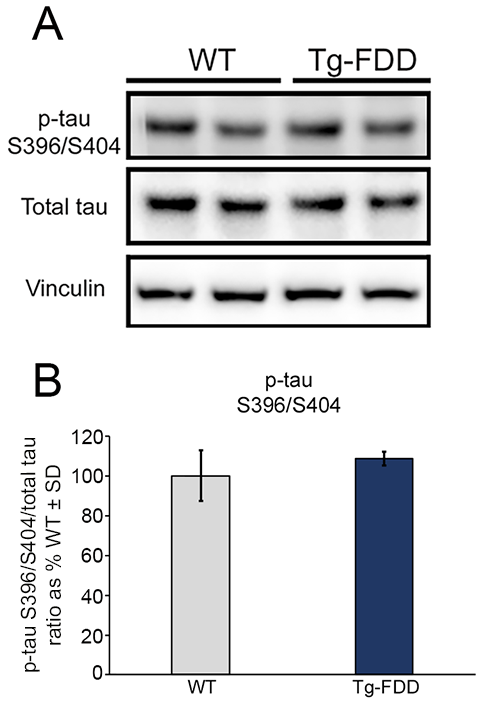


**Figure S5: Young Tg-FDD mice do not show changes in tau. (A)** Western blot of brain from 3 months old WT and Tg-FDD mice. **(B)** Graph showing WB quantification of p-tau S396/S404.


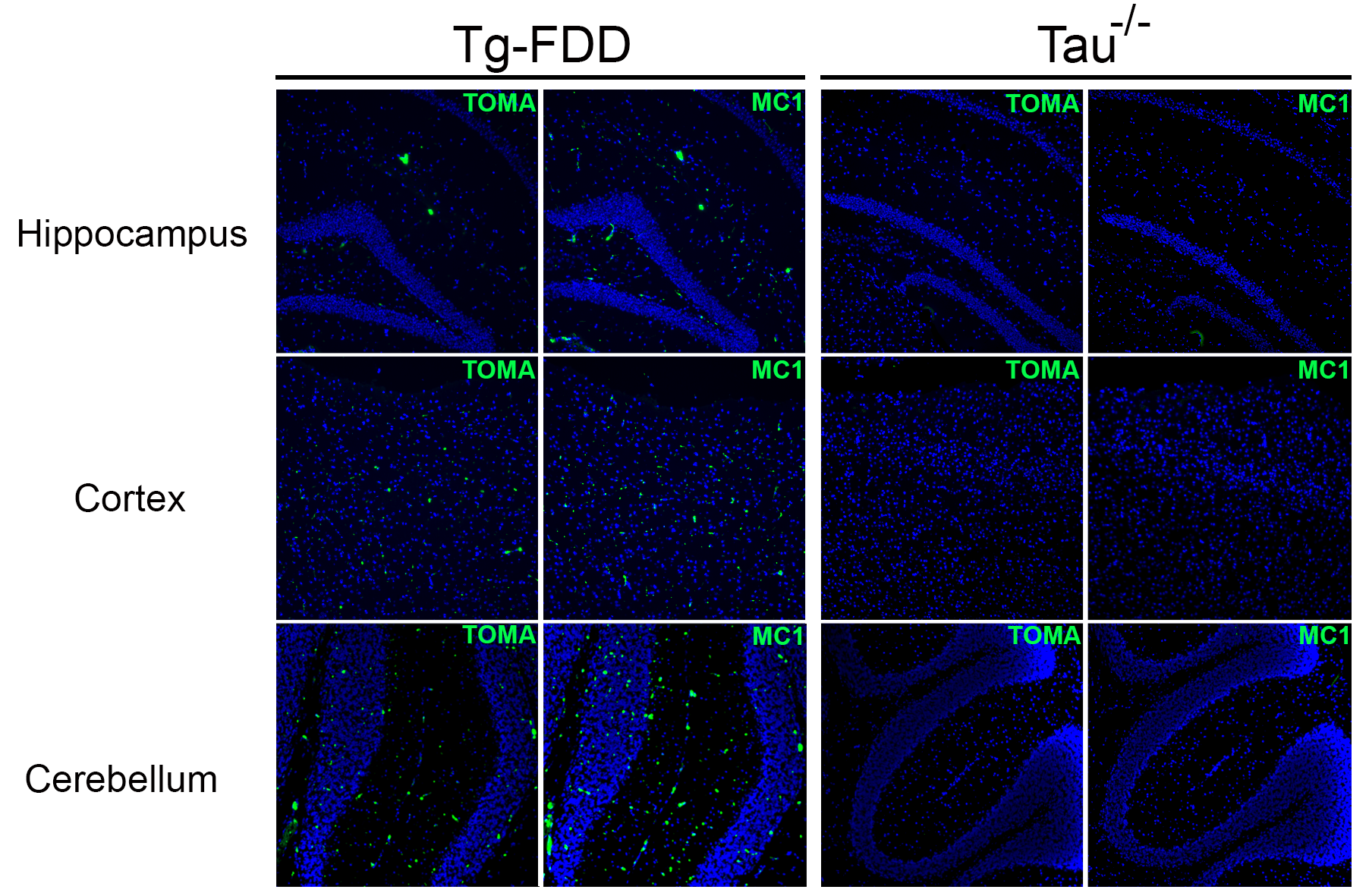


**Figure S6: Tau oligomers in Tg-FDD mice.** IF using the TOMA antibody (green) revealed the presence of tau oligomers in the hippocampus, cortex, and cerebellum of 18 months old Tg-FDD mice. Adjacent sections were stained with the MC1 antibody (green) that recognizes misfolded tau. MC1-positive staining was also observed in the hippocampus, cortex, and cerebellum of these mice. DAPI (blue) was used to stain the nucleus and Tau^-/-^ was utilized as a negative control.


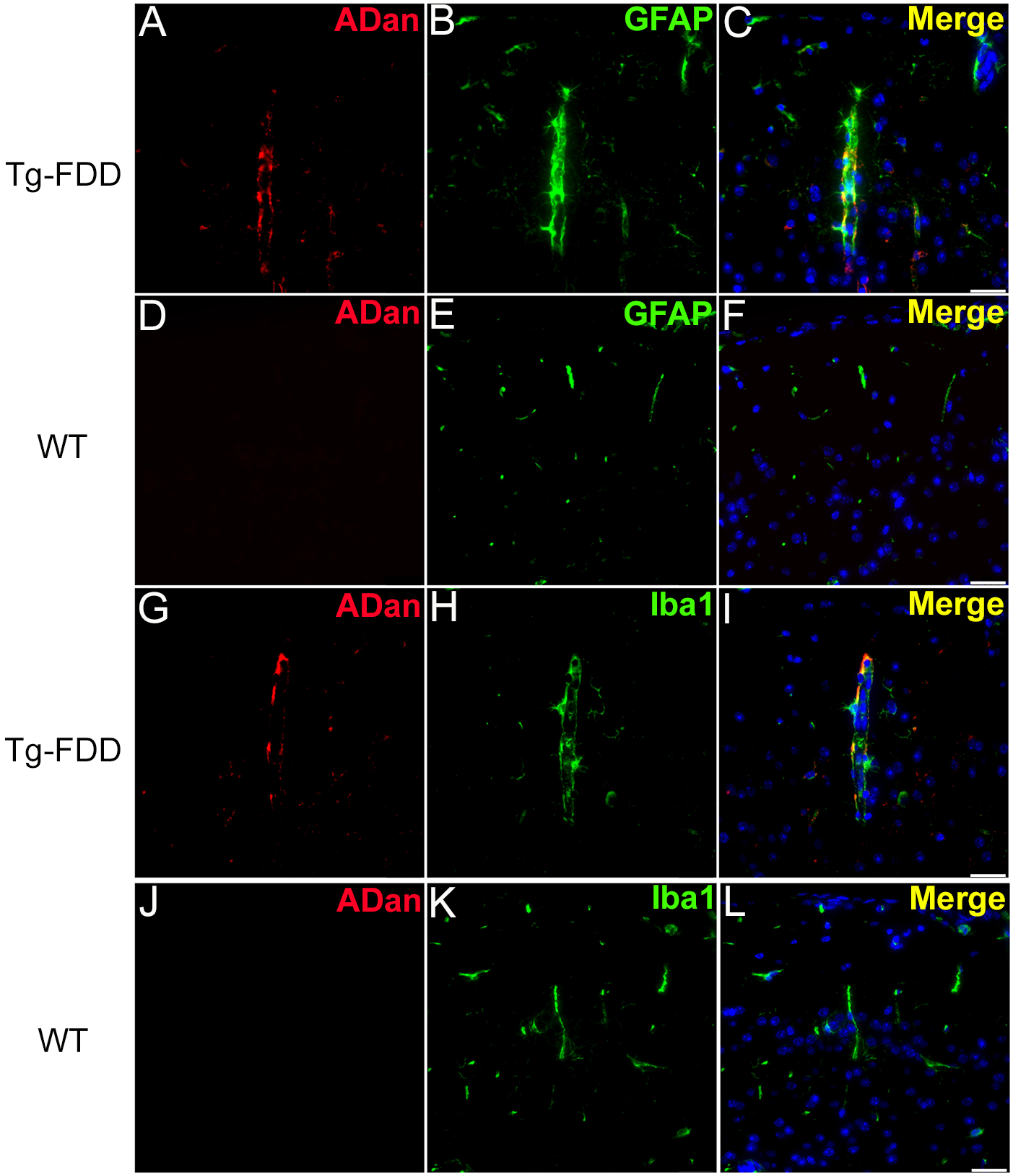


**Figure S7: Glial activation associated to CAA. (A-F)** IF of ADan amyloid (red) and GFAP (green) in Tg-FDD (A-C) and WT (D-F). **(G-L)** IF of ADan amyloid (red) and Iba1 (green) in Tg-FDD (G-I) and WT (J-L). Scale bar 25 µm.
